# The molecular and cellular basis of gonadal sex reversal in mice and humans

**DOI:** 10.1002/wdev.42

**Published:** 2012-02-28

**Authors:** Nick Warr, Andy Greenfield

**Affiliations:** Mammalian Genetics Unit, MRC HarwellOxfordshire, UK

## Abstract

The mammalian gonad is adapted for the production of germ cells and is an endocrine gland that controls sexual maturation and fertility. Gonadal sex reversal, namely, the development of ovaries in an XY individual or testes in an XX, has fascinated biologists for decades. The phenomenon suggests the existence of genetic suppressors of the male and female developmental pathways and molecular genetic studies, particularly in the mouse, have revealed controlled antagonism at the core of mammalian sex determination. Both testis and ovary determination represent design solutions to a number of problems: how to generate cells with the right properties to populate the organ primordium; how to produce distinct organs from an initially bipotential primordium; how to pattern an organ when the expression of key cell fate determinants is initiated only in a discrete region of the primordium and extends to other regions asynchronously; how to coordinate the interaction between distinct cell types in time and space and stabilize the resulting morphology; and how to maintain the differentiated state of the organ throughout the adult period. Some of these, and related problems, are common to organogenesis in general; some are distinctive to gonad development. In this review, we discuss recent studies of the molecular and cellular events underlying testis and ovary development, with an emphasis on the phenomenon of gonadal sex reversal and its causes in mice and humans. Finally, we discuss sex-determining loci and disorders of sex development in humans and the future of research in this important area. *WIREs Dev Biol* 2012, 1:559–577. doi: 10.1002/wdev.42

## INTRODUCTION

### Sex-Determining Gene Regulatory Networks

Gonadal sex reversal is a developmental phenomenon that reveals key features of the sex determination process in mammals. The formation of an ovary in an XY embryo, or a testis in an XX embryo, due to a specific genetic abnormality is reminiscent of a homeotic transformation. Homeosis describes the replacement of one embryonic structure by another^1^—but, crucially, this second structure is normally found elsewhere in the embryo, as in the famous Antennapedia mutations of *Drosophila melanogaster* that switch segment identity to such dramatic effect. Gonadal sex reversal is similarly the transformation of one structure into another normally found elsewhere—in this case, in the opposite sex. A homeotic mutation is no mere disruption to morphogenesis, and similarly, neither is gonadal sex reversal. Homeosis reveals how genetic programs can compete for dominance in an embryonic primordium and how the balance between such competing programs can be altered by loss or gain of gene function resulting in dramatic or subtle changes in developmental fate.^2^ Similarly, in recent years, it has become clear that the development of a testis or ovary from a bipotential primordium requires the controlled antagonism of one organogenetic pathway by another, in addition to the execution of a specific morphogenetic program. In this review, we will focus on those early events in mammalian (mouse and human) gonad development that, when disrupted, can lead to gonadal sex reversal and discuss what studies of these have taught us about the sex-determining mechanism in mammals and what remains to be discovered. We will also comment on sex reversal as an example of human birth defects and the genetic bases of such disorders of sex development (DSD). We will not discuss abnormalities of sexual development that do not originate in defects of sex determination and differentiation. Thus, cases of phenotypic sex reversal such as complete androgen insensitivity syndrome (CAIS) are not included.^3^

**FIGURE 1:**
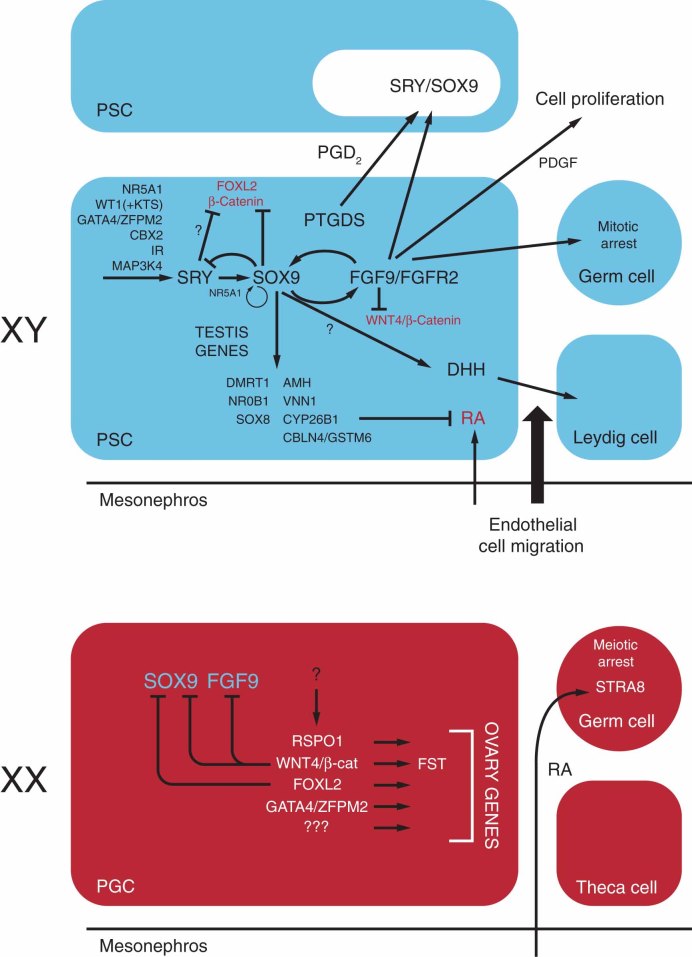
Model of gene regulatory networks in mammalian sex determination. Sex-determining genetic activity in the XY pre-Sertoli cell (PSC) is dominated by SRY-SOX9-FGF9/FGFR2. This genetic pathway is stabilized by mutually positive (curved arrows) interactions between SOX9 and FGF9, resulting in continued expression of SOX9 in the Sertoli cell lineage, in combination with antagonism (hammered lines) of ovary-determining genes/proteins (shown in red). Only a short period of SRY expression is required to upregulate *Sox9* from its basal levels in the bipotential gonad. FGF9 and PGD_2_ support paracrine signaling (arrows exiting from lower cell), resulting in the recruitment of additional supporting cell precursors (upper PSC) that express SRY and/or SOX9 to the Sertoli cell lineage. The secreted molecules FGF9 and desert hedgehog (DHH) also function in masculinizing the germ cell and steroidogenic cell lineages, respectively. This testis-determining gene regulatory network and its downstream targets (‘testis genes’) initiate a variety of morphogenetic processes, including the migration of endothelial cells from the adjacent mesonephros, which are crucial to the formation of testis cords and the associated coelomic blood vessel. In the female (XX) gonad, the absence of SRY in pregranulosa cells (PGC) results in successful antagonism of SOX9/FGF9 by ovary-determining gene products. This causes commitment to the granulosa cell lineage and permits retinoic acid (RA) to drive ovarian germ cells into meiosis. Note the linear core of the testis-determining pathway and the somewhat more modular appearance of the ovary-determining network. Note also that NR5A1 functions at distinct stages—this is likely to be true of several molecules included. Question marks indicate missing components or uncertainty in terms of regulatory relationships. IR = INSR, INSRR, and IGF1R.

**FIGURE 2:**
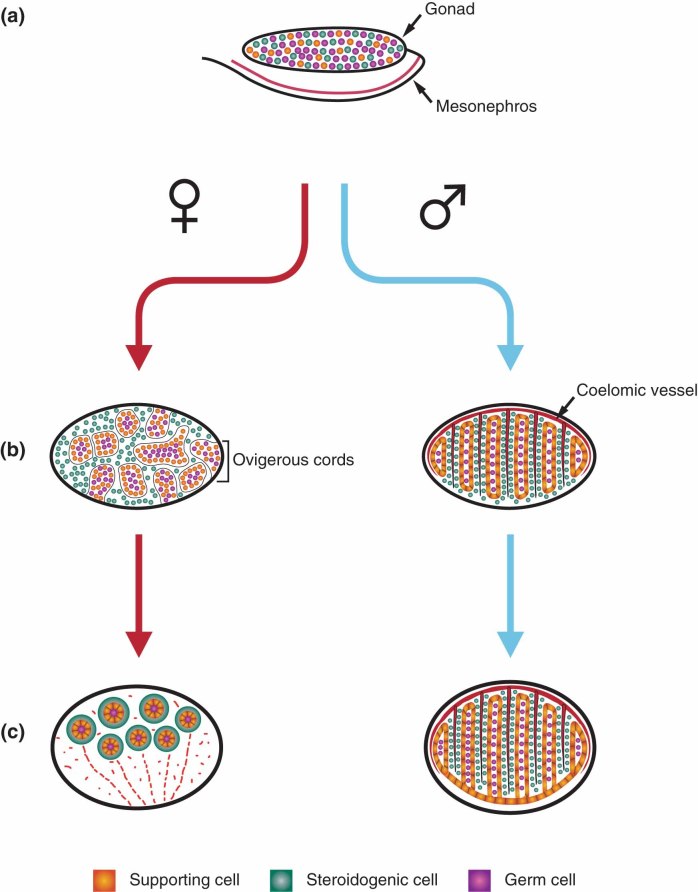
Cell lineages of the embryonic/fetal ovary and testis. (a) Color-coded diagram showing the arrangement of the bipotential somatic (supporting and steroidogenic) and germ cell lineages of the early gonad (around E11.5 in the mouse). The gonad forms on the ventromedial surface of the mesonephros. (b) In the female gonad, from around E13.5, germ cells and pregranulosa (supporting) cells are arranged into structures called ovigerous cords (oc), surrounded by interstitial mesenchymal cells (including steroidogenic precursors). These structures are not visible by light microscopy but can be revealed by immunostaining. In contrast, robust testis cords comprising germs cells and Sertoli cells (also supporting cells) are visible at a comparable stage in males, as is the prominent coelomic vessel (cv) that is formed from migratory mesonephric endothelial cells. Two interstitial cell types are prominent at this stage: peritubular myoid cells (not shown) that surround the testis cords and the steroidogenic Leydig cells, which produce androgens. (c) At around birth in the mouse, the ovigerous cords break down into primary follicles, containing a single oocyte surrounded by layers of granulosa cells and then theca (steroidogenic) cells.

We begin with a brief outline of our current understanding of testis and ovary determination at the molecular and cellular level (see overview in Figures 1 and 2). This framework will be used to focus on particular events in sex determination and flesh out some of the details of why disruption to these can cause sex reversal. In a review such as this, it is impossible to cover all topics that might be relevant: here, we will pay particular attention to the regulation of the fate of supporting cell precursors, the differentiation of pre-Sertoli cells or pregranulosa cells, as a key event in sex determination. The significance of this event is borne out by the function of known sex-determining genes and the sex reversal associated with their disruption.

The mouse gonad forms on the ventromedial surface of the mesonephros at around embryonic day (E) 10.0 (the term ‘E’ is used here in a way that is interchangeable with ‘days *post coitum*’). Somatic cells of the gonad are the site of the sex-determining ‘decision’: in XY embryonic gonads, a subset of these somatic cells in the central region of the gonad, which are bipotential, commit to becoming pre-Sertoli cells under the influence of the gene sex-determining region Y (*SRY*). In the absence of SRY, pregranulosa cells differentiate from this same lineage. The central role of *Sry* expression in this lineage is supported by numerous areas of research, such as analysis of the gonads of XX-XY chimeric embryos and *Sry*-reporter gene expression profiles (see below). SRY acts to upregulate the transcription of the related gene, *Sox9,* in a cell autonomous fashion. Once cellular- and tissue-level thresholds of *Sry* (and *Sox9*) expression are met (Box 1), SOX9 protein then acts to ‘lock-in’ its own expression such that it is subsequently maintained at high levels in the Sertoli cell lineage. This is achieved through its positive interaction with the secreted molecules fibroblast growth factor 9 (FGF9) and prostaglandin D2 (PGD2). These molecules are thought to be important in securing two other necessary steps in testis determination: the recruitment of other somatic cells to the Sertoli cell fate and, in the case of FGF9, spread of the initial central testis-determining signal to the anterior and posterior poles of the gonad. Once sufficient numbers of SOX9-positive cells are established throughout the gonad, the familiar morphogenetic program of testis differentiation ensues. This morphogenesis is a highly dynamic process, involving integration of a number of cellular processes including cell proliferation, endothelial cell migration from the mesonephros, differentiation of interstitial cell lineages (Leydig and peritubular myoid cells), epithelialization of Sertoli cells and their enclosure of germ cells, mitotic arrest of these germ cells, and the formation of testis cords and the associated coelomic blood vessel and tributaries thereof. Recent studies suggest that, while the differentiation of pre-Sertoli cells is the key event in initiating testis determination and development, the supporting cell lineage may not occupy center stage in subsequent morphogenetic processes but, rather, is one of a number of cell lineages whose interaction is required for testis cord formation.^4^,^5^ We will later address the question of whether disruption to processes other than supporting cell precursor differentiation might result in gonadal sex reversal. In females, the absence of SRY results in little overt morphological change over the first few days of gonadogenesis, primarily due to reduced growth and absence of mesonephric cell migration. Germ cells enter meiosis and by birth, these are associated with ovigerous cords, which subsequently fragment into primordial follicles (Figure 2).

**BOX 1 CELL PROLIFERATION AND THE REGULATION OF *SRY* EXPRESSION**

The requirement that a threshold of SRY-positive cells exist in the XY gonad in order for testis development to proceed, a fact established by analysis of gonad development in XX-XY chimeras, strongly suggests that proliferation of Sertoli cell precursors may be an important component of testis determination. The rapid growth of the male gonad also supports such a role for cell proliferation. Studies of cell proliferation in the developing gonad have established that a male-specific, SRY-dependent increase in cell proliferation occurs in XY gonads compared to XX gonads from approximately E11.2 to E11.8, an effect concentrated in NR5A1-positive cells of the coelomic epithelium.^35^ Moreover, pulse-chase experiments aimed at determining the fate of proliferating cells indicated that if dividing cells were labeled prior to E11.5, these contributed to the Sertoli cell population. This was not the case if labeling occurred after E11.5. Such observations naturally lead to the conclusion that the observable difference in proliferation at the coelomic epithelium is a requirement for testis determination, presumably by establishing a sufficiently large population of Sertoli cell precursors. This conclusion is reinforced by lineage tracing experiments using DiI labeling. In these studies, cells labeled at the coelomic epithelium from E11.2 to E11.4 moved into the gonad and contributed to the Sertoli cell lineage.^36^ However, a subsequent study revealed that XY gonads frequently lacked testis cords and the Sertoli cell marker, *Sox9,* when treated with proliferation inhibitors from approximately E10.8 to E11.2.^37^ Inhibition before or after this window did not disrupt testis development. Thus, while these studies established the importance of cell proliferation in the XY gonad for testis development, they indicated that detectable differences in cell proliferation between XX and XY at the coelomic epithelium around E11.5 are probably not a requirement for testis development but more likely a consequence.^37^ Lineage tracing studies suggest that the coelomic epithelium is indeed an important source of Sertoli cell precursors, but the cells that are sensitive to the action of proliferation inhibitors remain to be formally identified.

The contribution of reduced numbers of cells expressing *Sry* versus reduced expression in each cell has not been addressed in many cases of XY sex reversal caused by loss of *Sry* expression. In the case of embryos lacking the +KTS isoform of *Wt1*^38^ and *Map3k4*,^30^ reduced expression of SRY in individual cells is also associated with reduced numbers of SRY-positive cells. This outcome is predicted by models that offer an account of SRY expression that depends on appropriate levels of functional SRY at the cellular level, leading to recruitment of additional SRY-positive cells.^38^,^39^ Whether defects in cell proliferation alone can lead to small numbers of cells expressing normal levels of SRY remains to be seen. However, models of SRY expression that depend on functional SRY appear to conflict with the reported expression profile of a non-functional enhanced green fluorescent protein (EGFP) *Sry* reporter transgene.^16^ *Sry*-EGFP reporter expression broadly mimicked that of endogenous *Sry*, with the exception that expression was also observed in XX gonads. Up until approximately E12.0, the temporal and spatial expression of EGFP was essentially identical in XY and XX gonads. These data indicate that, at least up until the initiation of testis cord formation, generation of the normal profile of *Sry* expression does not require functional SRY. Further study of this and other reporter constructs may be necessary to answer this question definitively, and more detailed examination of the mechanisms by which *Sry* expression is controlled are clearly required.

Crucially, SOX9 actively promotes testis determination, presumably by activation of a range of pro-testis genes, and suppresses the ovarian pathway of development. How is such an antagonistic interaction achieved? It is thought that molecular determinants of ovary differentiation, such as the canonical wingless-related MMTV integration site (WNT) signaling pathway and the transcription factor, FOXL2, are directly or indirectly antagonized by SOX9 and potentially other pro-testis gene products. However, the antagonism is mutual. For example, *β*-catenin is thought to antagonize SOX9 by direct physical interaction, as it does in other developmental contexts. Moreover, genetic evidence in mice indicates a role for FOXL2 in repression of *Sox9* expression in ovarian somatic cells in adult life. In the absence of SRY, *Sox9* expression in the early gonad remains at very low levels and is eventually lost. The pro-ovarian genes and pathways mentioned above are then able to execute ovary developmental programs, resulting in the differentiation of granulosa cells and theca cells and the entry of germ cells into meiosis. The ovarian pathway and its complexities will be discussed in more detail later.

**FIGURE 3:**
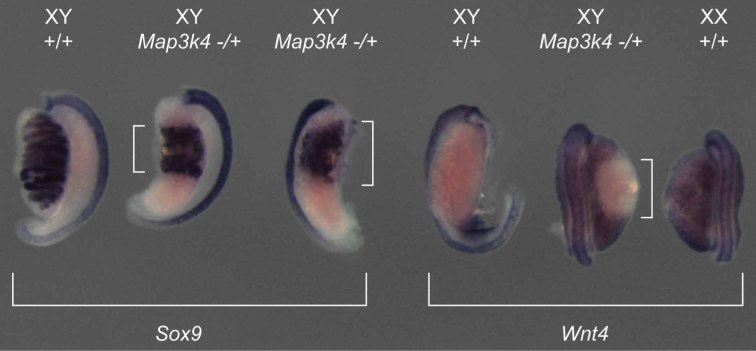
Ovotestis development in embryos haploinsufficient for *Map3k4.* Ovotestis development (previously known as true hermaphroditism) is associated with partial disruption to the testis-determining pathway. The examples from the mouse shown here are caused by a combination of three genetic factors: haploinsufficiency (−/+) for the gene *Map3k4*, a C57BL/6J (B6)-derived set of autosomes and X chromosome, and a Y chromosome from the AKR strain (Y^AKR^). The wild-type (+/+) B6 Y^AKR^ gonads have ordered stacks of testis cords (around 10), highlighted by the expression of the Sertoli cell marker, *Sox9*, and absence of the ovarian somatic marker, *Wnt4*. Ovotestes are characterized by regions of testicular tissue (brackets) that are immediately adjacent to ovarian tissue in the same gonad (revealed by marker gene expression). The ovarian regions of the ovotestis are usually at one or both poles. It is thought that the sensitivity of the gonadal poles to local sex reversal is caused by a delay in the receipt of the masculinizing signal (thought to include FGF9) that emanates from the gonad center earlier in development (see text).

This mutual antagonism at the molecular level seems likely to reinforce, or canalize, the consequences of the initial sex-determining events. The fascinating phenomenon of ovotestis development aside (see below and Figure 3) the newly formed gonad is strongly committed to one of two possible developmental outcomes, and nothing in-between. The existence of a bipotential gonadal primordium presumably increases the risk that divergent genetic programs, both of which are natural to that primordium, might attempt to ‘execute’ at the same time, with disastrous consequences. Thus, an antagonism that acts to minimize this possibility makes evolutionary ‘sense’.

With this background in place, we will now focus on instances of gonadal sex reversal in mammals in order to shed light on particular features of sex determination that are revealed by them. These discussions will permit us to address some unanswered questions and will be structured around some of the major events described in this section.

## SPECIFYING THE FATE OF SUPPORTING CELL PRECURSORS

### SRY: Protein Function and Regulation of Gene Expression

There has been a good deal of discussion of *SRY* and its idiosyncratic properties (reviewed in Ref 6). This 'weak, ‘hesitant’, ‘lazy’ gene, marooned on the nonrecombining portion of the Y chromosome and suffering mutational degradation as a consequence, is barely fit for purpose, apparently. In the mouse, it is expressed briefly (for approximately 6–8 h in a given somatic cell) and at low levels. Its function is notoriously sensitive to disruptions to the timing or level of its expression. In this section, we will review sex-reversing mutations that are thought to impact on the testis-determining pathway by impacting on *SRY* expression and protein function.

#### The Molecular Function of SRY

SRY is a high-mobility group (HMG) domain- containing protein that binds and bends DNA. Recently, it has been established that it binds to a testis-specific enhancer sequence (TESCO) upstream of the *SOX9* gene and, in concert with NR5A1 (formerly known as SF1), activates its transcription.^7^ It is unclear whether any other *bona fide* targets of SRY exist in mice or humans (though targets are regularly reported^8^), or whether SRY also has functions independent of its DNA-binding capacities, but the role it plays in upregulating *SOX9* transcription is sufficient to explain its requirement for testis determination. The fact that most human XY females with *SRY* mutations have these in the HMG domain, and that the protein is poorly conserved outside of this domain, supports the idea that its testis-determining role is best described as the timely provision, transiently in the mouse, of an appropriate HMG box domain for the upregulation of *SOX9* transcription in Sertoli cell precursors. This model is also supported by the discovery that, in mouse transgenic models, the inappropriate expression of alternative SOX proteins at early stages of XX embryonic gonad development is also sufficient to activate *Sox9* expression and induce testis development.^9^,^10^ Regulatory mutations that affect the expression of additional SOX genes may, therefore, account for some instances of XX male development in the human population.

#### The Dynamic Profile of Sry Expression

The expression profiles of *Sry* and its protein product in the developing mouse gonad have been the subject of intensive study using a number of assays.^11^–^20^ Historically, this focus has been motivated by a number of questions concerning testis determination, including the gonadal cell lineage in which testis determination is initiated, the molecular basis of the timing and threshold phenomena mentioned earlier, the cause of ovotestis development, and the regulatory relationship between *Sry* and other genes. Studies utilizing RNA *in situ* hybridization and immunodetection have revealed a dynamic profile of expression, with a center-to-pole (CP) expansion over time.^17^,^20^ *Sry* transcription is initiated in the central region of the mouse gonad at around E10.75 [12 tail somites (ts)]. Small numbers of cells expressing SRY protein can be detected centrally at the same stage, close to the coelomic epithelium.^18^ Over the next 18 h or so, *Sry* expression spreads to the poles and is expressed throughout the gonad by E11.5 (18 ts). Expression is also extinguished in a spatially and temporally complex fashion, first at the center of the gonad and then at the anterior and, finally, posterior pole of the gonad. *Sry* is undetectable by E12.5. Interestingly, two markers of pregranulosa cells, *Sprr2d* and *1700106J16Rik*, have also been reported to have a similar CP expression profile.^21^ This suggests possible shared regulatory mechanisms in the control of expression of *Sry* and these two genes during establishment of the supporting cell precursor lineage in XY and XX gonads. It is also a reminder of the common developmental origins of the male and female gonadal supporting cell precursors.

The tightly orchestrated and dynamic cellular and tissue profile of *Sry* expression in the mouse gonad is inextricably bound up with the idea that it functions in a narrow window of opportunity to induce commitment to the Sertoli cell fate.^22^ A number of incidences of XY gonadal sex reversal in the mouse depend on the presence of a *Mus domesticus* Y chromosome and the C57BL/6J genetic background: so-called B6.Y^DOM^ sex reversal. In C57BL/6J-Y^*POS*^ (B6.Y^*POS*^) sex reversal, varying degrees of ovarian tissue develop in XY individuals when autosomal loci are homozygous B6 and the *Sry* allele is derived from *Mus domesticus poschiavinus*.^23^ Affected XY individuals either develop two ovaries, an ovary and an ovotestis, or two ovotestes. Ovotestes are characterized by the formation of testicular tissue at the center of the gonad and ovarian tissue at one or both poles (Figure 3). Studies of gene expression in the gonads of B6.Y^*POS*^ embryos suggest that delayed and reduced expression of the testis-determining genes *Sry* and *Sox9* are the likely cause of sex reversal or ovotestis formation.^19^ The failure of such testis-determining genes to completely suppress the ovarian genetic pathway, especially at the poles of the B6.Y^*POS*^ gonad, is thought to underlie ovotestis development.^24^ Because the gonadal poles are the last to receive the testis-determining signal, it appears that they are most prone to any delay in *Sry* expression, and consequent failure to sufficiently upregulate *Sox9*, and thus the local sex-reversing effects of failing to preempt the ovary-determining pathway. The precociousness of this ovarian pathway depends on genetic background.^25^ Regionalized sex reversal in ovotestes highlights the importance of both cell-autonomous and non-cell-autonomous functions during mammalian sex determination. Interestingly, Y chromosome deletions in the vicinity of the *Sry* locus, but not including the gene itself, have also been shown to reduce *Sry* expression and result in XY female development or hermaphroditism, again highlighting the requirement for *Sry* to be expressed in a timely fashion and to reach a threshold level.^12^ The location of *Sry* on the largely heterochromatic Y chromosome may be an important factor in the regulation of its expression.

Targeted deletion and/or mutation of a number of genes in the mouse, either individually or in combination, is reported to cause XY sex reversal by disruption to *Sry* expression, although the severity of the disruption to testis determination is also background dependent in these cases and most severe on B6. These genes include *Cbx2* (formerly known as *M33*),^26^ the +KTS isoform of *Wt1,*^27^ *Gata4*,^28^ *Zfpm2* (formerly known as *Fog2*),^28^ *Nr5a1,*^29^ *Cited2,*^29^ and *Map3k4*.^30^ A triple knockout of *Insr* (formerly known as *Ir*)*, Insrr* (formerly known as *Irr*), and *Igf1r* also causes XY sex reversal by impacting negatively on *Sry* expression.^31^ Reduced dosage, rather than complete deletion, of some of these genes is also associated with ovotestis development. Moreover, homologs of several of these genes are implicated in human testis determination. It is tempting to place these loci ‘upstream’ of *Sry* in a pathway. Several of the genes in question encode transcription factors, cofactors, or chromatin factors that might play a direct role in transcriptional regulation of *Sry*; others, such as *Insr/Insrr/Igf1r* and *Map3k4,* do not fit simply into such a transcriptional model and may function in recruitment to the pre-Sertoli cell lineage, or both processes. Again, it is important to account for the reduction in *Sry* levels in these mutants: are cells that normally express *Sry* still doing so, but at reduced levels? Or has the total number of *Sry*-positive cells been reduced as a consequence of the mutation? See Box 1 for more details on this topic.

In addition to delayed *Sry* expression, some reports have indicated that failure to subsequently repress *Sry* transcription is also associated with disruption to testis determination.^32^ SOX9 has been implicated in the downregulation of *Sry*, primarily due to persistent *Sry* expression in gonads lacking SOX9,^33^,^34^ although this association does not hold in all contexts.^24^ SRY expression is more widespread in other mammalian species, and the functional significance of the spatiotemporally narrow, gonad-specific expression profile in the mouse embryo remains unclear. What does seem clear is that the primary function of SRY is to upregulate *Sox9* and that delayed or reduced *Sry* expression can cause XY sex reversal by failure to boost *Sox9* expression and thereby specify the Sertoli cell fate.

### *SOX9*—Master Testis-Determining Gene

*SOX9*, which, like SRY, encodes a transcription factor containing an HMG domain, is thought to be a protestis gene in all vertebrates. Mutations of *SOX9* cause XY gonadal sex reversal in humans and mice, and the gene is expressed during testis development in fish, amphibians, reptiles, and birds—and prior to sex determination in examples from the latter two classes. Moreover, analysis of highly evolutionarily conserved regions of the TESCO gonadal enhancer of *SOX9* in multiple species has led to a model whereby distinct transcription factors in species with distinct chromosomal sex-determining mechanisms might act through these conserved enhancer sequences to upregulate *SOX9* expression and ultimately permit autoregulation and maintenance of expression in the gonad.^40^ Cell line data also support a role for the human TES homolog in SRY-, NR5A1-, and SOX9-mediated upregulation of *SOX9* transcription and disruption to this in cases of 46,XY DSD.^41^ Thus, at the very least, it seems likely that SOX9 plays a major conserved role in testis determination in a number of vertebrate species.

In the mouse, *Sox9* is initially detected in XY and XX gonads at around E10.5 before being upregulated sex-specifically at E11.5 in XY pre-Sertoli cells^42^,^43^; it is maintained at high levels thereafter, in stark contrast to *Sry* whose expression is transient. Nuclear translocation of SOX9 is thought to be important for its male-specific activities at this stage. Loss of SOX9 is associated with complete XY gonadal sex reversal in humans and mice due to failure of Sertoli cell differentiation.^33^,^34^,^44^–^46^ Moreover, forced expression of *Sox9* in embryonic mouse XX gonads results in testis development,^47^,^48^ and duplication of *SOX9* in human XX individuals has been reported to cause gonadal sex reversal.^49^,^50^ Thus, *SOX9* is both necessary and sufficient for testis determination. Curiously, despite its prolonged expression in supporting cells, SOX9 does not appear to be required for somatic testis development after E14.5 in mice, possibly due to redundant functions of the related protein, SOX8.^51^,^52^ However, a role in suppressing the reprogramming of adult Sertoli cells has been described for DMRT1. Deletion of *Dmrt1*, during XY fetal development and in adults, results in reprogramming of differentiated Sertoli cells in the postnatal testis into granulosa-like cells that express *Foxl2* and other ovary-enriched genes.^53^ Indeed, Sertoli cell-specific deletion of *Dmrt1* resulted in feminized XY gonads containing germ cells arrested in meiotic prophase.^53^

The regulation of *Sox9* expression requires the intervention of two additional testis-determining genes: *Sry* and *Fgf9*. Recent data in the mouse have demonstrated a cell autonomous role for SRY in the upregulation of *Sox9* transcription by binding to its gonadal ridge enhancer, TESCO.^7^,^18^ However, analysis of human TESCO sequences in 66 cases of 46,XY gonadal dysgenesis (GD; Swyer syndrome) revealed no mutations in two cohorts, suggesting that TESCO mutations are not a common cause of 46,XY GD in humans.^54^ In mouse, SRY cooperates with NR5A1 in binding to TESCO. However, as its brief period of expression suggests, SRY is required only to initiate the high-level transcription of SOX9 characteristic of Sertoli cells. Maintenance of SOX9 expression requires both cell-autonomous and extracellular signaling elements. First, SOX9 is capable of binding TESCO and, again in concert with NR5A1, activating its own transcription.^7^ Second, genetic evidence suggests a role for fibroblast growth factor 9 (FGF9) in maintenance of *Sox9* transcription. Analysis of embryonic gonads from embryos lacking *Fgf9* reveals normal upregulation of *Sox9*, but a failure to maintain high levels after E11.5.^55^ Moreover, *Fgf9* transcription is disrupted in embryonic gonads lacking *Sox9*.^55^ These data suggest a genetic pathway in which *Sox9* and *Fgf9* activate each other in an interaction involving positive feedback. How this interaction occurs at the molecular level is unclear. *SOX9* is a gene with a notoriously complex control region, extending over a megabase in humans, and this does not assist in identifying genuine regulatory interactions. The role of FGF9 and another secreted molecule, prostaglandin D_2_ (PGD_2_), in regulating *Sox9* expression and propagating the masculinizing signal initiated by SRY will be discussed in the next section. These molecules act to ensure that SOX9 is expressed at high levels in cells of the supporting cell lineage and that sufficient numbers of *SOX9*-expressing cells are recruited. In addition to secreted signaling molecules, other transcription factors have been implicated in mouse *Sox9* regulation. Ablation of *Wt1* at around E14.5 in embryonic XY gonads results in loss of *Sox9* expression and progressive loss of Sertoli cells, suggesting that WT1 has a role in regulating *Sox9* expression, either directly or indirectly, possibly by inhibition of *β*-catenin.^51^,^56^ Finally, there are reported roles for NR0B1 (formerly known as DAX1), GATA4, and ZFPM2 in regulation of *Sox9* expression and Sertoli cell differentiation.^57^–^60^

It seems likely that SRY acts to regulate one transcriptional target: *Sox9*. This positive regulation is a requirement for Sertoli cell differentiation. Ectopic expression of *Sox9* in developing mouse XX gonads can mimic the effect of *Sry*,^47^,^48^ but no other downstream gene has been reported to do the same. What are the transcriptional targets of SOX9 itself and how do these molecules secure Sertoli cell differentiation? As Figure 1 indicates, the pathway of testis determination extends multiple branches at this juncture. Excluding *Sox9* and *Fgf9*, whose functions appear to center on the establishment of the feedback loop described above, none of the known targets of SOX9 transcriptional regulation, such as *Ptgds* (whose product generates PGD_2_; see below),^61^ *Vnn1*,^62^ and *Amh*,^63^ are themselves testis-determining genes, as evidenced by the absence of gonadal sex reversal upon their deletion. To this extent, it appears that *SOX9* is not only the primary mammalian testis-determining gene (whose function, unlike SRY, cannot be readily replaced by another molecule) but also the *last* such gene, in the sense that molecules acting downstream appear to execute only subroutines in the network of molecular activities required for testis determination. No doubt, given the complexity of the functional specialization of the Sertoli cell itself, additional targets of SOX9 remain to be identified.

### Extracellular Signaling and Non-cell Autonomous Functions in Testis Determination

A role for fibroblast growth factors (FGFs), a family of extracellular signaling factors, in testis determination was established when mice lacking *Fgf9* were shown to exhibit XY gonadal sex reversal.^64^ This was associated with disruption to Sertoli cell differentiation, cell proliferation, and mesonephric cell migration, all early features of testis determination.^65^ *Fgf9* is initially expressed in XX and XY gonads but becomes restricted to the male gonad by around E11.5.^55^,^66^ Once testis cords have formed, *Fgf9* expression is restricted to Sertoli cells, suggesting a role in specifying the fate of supporting cell precursors at early stages. Studies at E11.5 revealed that *Sry* expression is not dependent on FGF9.^55^ Moreover, initial analysis of *Sox9* expression in XY *Fgf9* −/− gonads revealed that initiation and upregulation of *Sox9* expression at E11.5 was normal. However, SOX9 protein was not detectable in mutant gonads by E12.5, suggesting that FGF9 plays a role in the maintenance of SOX9 expression. As described earlier, the relationship between *Sox9* and *Fgf9* was shown to be mutually reinforcing: *Fgf9* expression was also significantly decreased or absent in XY gonads lacking *Sox9* at E11.5, suggesting the existence of a positive feedback loop between the two loci that drives testis determination. In addition to its positive role in the regulation of *Sox9* expression, high levels of the ovarian marker, *Wnt4,* in XY *Fgf9* −/− gonads at E11.5 suggest that *Fgf9* specifically antagonizes *Wnt4* expression. Moreover, *Fgf9* is expressed in XX *Wnt4* −/− gonads at E12.5, in contrast to XX *Wnt4* −/+ controls. Thus, *Fgf9* and *Wnt4* expression represent opposing signals in the determination of gonadal sex.^55^

The receptor for FGF9 during testis determination was postulated to be FGFR2 based on expression studies of distinct FGF receptors: FGFR2 was detected at the plasma membrane of coelomic epithelial cells and in the nucleus of pre-Sertoli cells.^65^ This hypothesis was essentially confirmed when varying degrees of XY gonadal sex reversal were reported after gonad-specific deletion of *Fgfr2*.^67^,^68^ The use of distinct *Cre* deleter lines and a floxed *Fgfr2* allele revealed a role for FGFR2 in cell proliferation and Sertoli cell differentiation, although deleting genes at high efficiency in all cells of a specific cell lineage of the developing gonad is challenging and this can make interpretation difficult. The role of cell proliferation in testis determination is discussed in more detail in Box 1.

Paracrine signaling by FGF9/FGFR2 might explain non-cell-autonomous events in testis determination, such as the recruitment of XX somatic cells to the Sertoli cell lineage in XX-XY chimaeras.^69^ A series of elegant organ culture experiments has revealed a role for FGF9 as a diffusible molecule that directs a CP expansion of tubulogenesis by its ability to stabilize *Sox9* expression.^70^ On the basis of these studies, the role conceived for FGF9 is one of a rapidly moving enforcer of the masculinizing signal that is initially restricted to the center of the XY gonad at around E10.75–E11.0. Despite this spatial restriction, there is no appreciable delay in the formation of testis cords at the poles of the XY gonad when compared to the center. Thus, FGF9 is thought to act as a diffusible signal that acts to rapidly pattern a long gonadal primordium. A degree of disruption to the core testis-determining genetic pathway (SRY-SOX9-FGF9) can lead to insufficient levels of FGF9 to rapidly establish high levels of SOX9 at the gonadal poles, resulting in a preemptive ‘strike’ by the ovary-determining pathway in these regions and the formation of an ovotestis. Of course, major disruption to the core pathway can, in the right genetic background, lead to a failure to establish sufficient levels of SOX9 required for Sertoli cell differentiation anywhere in the XY gonad, leading to complete gonadal sex reversal. PGD_2_ can also recruit SOX9-positive cells in a non-cell autonomous fashion and thereby reinforce the spread of the testis-determining signal.^20^,^71^ PGD_2_ signaling promotes protein kinase catalytic subunit alpha (PRKACA; formerly known as PKA)-dependent phosphorylation of SOX9 and KPNB1 (ß-importin 1)-dependent nuclear import in cell line models.^71^,^72^ Moreover, sex-reversing mutations in human SRY and SOX9 are thought to disrupt protein–protein interactions required for appropriate nuclear import, suggesting that the proper regulation of subcellular localization of these proteins, by posttranslational modification and protein interactions, is key to their testis-determining functions (reviewed in Ref 73). FGF9 and PGD_2_ may underlie protestis reinforcement mechanisms that are important due to the brief period of SRY expression and its sensitivity to disruption to the timing and levels of its expression, not least when that expression is initially restricted to one gonadal region. Other reinforcing mechanisms may yet be identified. Why SRY expression is initially restricted to cells at the center of the XY gonad is unclear, but this may be related to a prior restriction on the permissible routes by which some precursor cells migrate into the gonad from the coelomic epithelium. Further studies on gonad formation may shed light on such questions.

### Ovary-Determining and Antitestis Genes

The existence of an antitestis factor as a component of ovary development was first inferred from the frequency of XX males in the human population that are SRY negative.^74^ The simplest genetic explanation of such instances of XX sex reversal proposed the existence of a negative regulator of testis development, Z, which when disrupted due to loss of function mutations resulted in XX male development of varying degrees. Moreover, it was predicted that SRY would itself act genetically to inhibit Z. The prescience of these interpretations is borne out by subsequent investigations of sex determination in humans and mice, notwithstanding the complexity of antitestis and ovarian-promoting pathways in the female gonad.

#### Canonical WNT Signaling and Ovary Development

WNT4 is a secreted glycoprotein that was first associated with a role in XX gonad development due to its acting as a genetic suppressor of events associated with testis development.^75^,^76^ *Wnt4* is initially expressed in somatic cells of both sexes but is upregulated in ovarian somatic cells and downregulated in the XY gonad at E11.5. Loss of *Wnt4* resulted in masculinization of the XX gonad, including the formation of a coelomic blood vessel and the inward migration of adrenal steroidogenic cells capable of producing androgenic enzymes that masculinize the male reproductive tract primordia. However, germ cells enter oogenesis before subsequently degenerating. Moreover, gonadal supporting cells acquire features reminiscent of the testis, such as cord-like arrangements, only at birth and incompletely.^75^,^77^ Mutant embryos die at this stage due to kidney agenesis. Thus, gonadal sex reversal in *Wnt4*-deficient embryos is partial and the time-course of masculinization of different ovarian lineages is distinct and occurs late in the case of the supporting cells. These are not quite the properties predicted of the antitestis factor, Z.

A role for WNT4 in ovary development implicated canonical WNT signaling more broadly in sex determination. Subsequently, two additional molecules relevant to this signaling cascade were shown to be required for normal ovary development. Homozygosity for mutations in *RSPO1,* which encodes a secreted protein that activates WNT/*β*-catenin signaling, causes female to male sex reversal in XX humans^78^ (see also section on *46,XX Testicular Disorder of Sex Development*). Studies of *Rspo1* expression in mice revealed that it is predominantly expressed in XX gonadal somatic cells from E11.5. Expression in the male gonad at this stage is mainly restricted to the coelomic epithelium. As would be predicted from the presence of an activator of WNT/*β*-catenin signaling, transcriptional targets of *β*-catenin are specifically activated in somatic cells of XX gonads. Targeted disruption of *Rspo1* results in variable degrees of XX ovotestis development.^79^,^80^ As in *Wnt4*-deficient XX gonads, an ectopic coelomic blood vessel forms in mutant XX gonads by E12.5. However, these gonads are smaller than XY controls at the same stage and lack testis cords. By E18.5, XX *Rspo1* −*/−* gonads contained seminiferous tubules containing only a few gonocytes; but sex reversal was partial, and occasional nests of pachytene oocytes were also visible in some regions. The partial nature of the gonadal sex reversal, reminiscent of *Wnt4*-deficient gonads, was also apparent by marker gene expression analysis of mutant embryonic gonads. *Wnt4* levels were dramatically reduced from E11.5, suggesting a role for RSPO1 in upregulating *Wnt4* expression. But the testicular markers *Fgf9* and *Ptgds* were elevated at E14.5 and E18.5, though only slightly at earlier stages. *Sox9* expression at early stages was also weak. Low levels of SOX9-FGF9 may explain the hypoplastic gonads and the delay in masculinization of the mutant XX gonad. Ectopic expression of a stabilized form of *β*-catenin rescued the gonadal sex reversal, establishing that RSPO1 acts to suppress testis development through activation of *β*-catenin signaling.^79^ The antitestis properties of stabilized *β*-catenin were subsequently demonstrated by ectopic expression in developing XY gonads—this disrupted the male program and caused male-to-female sex reversal.^51^,^81^ The mechanistic basis of this antagonism is unclear, but a negative interaction between SOX9 and *β*-catenin, involving the degradation of heterodimers, has previously been described during chondrogenesis.^82^ In addition to gain-of-function studies, loss-of-function analyses confirmed a female-specific role for *β*-catenin in sex determination, with the identification of masculinized ovaries in *β*-catenin-deficient embryos, again reminiscent of *Rspo1*- and *Wnt4*-deficient ovaries.^83^

#### FOXL2 and the Maintenance of the Ovarian Soma

The partial gonadal sex reversal caused by loss of *Wnt4*, *Rspo1*, and *β*-catenin (*Ctnnb1*) suggests that the ovarian determination pathway in murine XX gonads is not a simple linear cascade analogous to the SRY-SOX9 axis in developing XY gonads. (Although the identification of a mouse genetic background that is especially sensitive to disruptions to ovary determination, if it exists, might force us to modify this conclusion somewhat.) The activation of canonical WNT signaling is required for suppression of the testis-determining pathway, but other factors appear to act autonomously to promote commitment to the ovarian pathway even in the absence of the WNT4/RSPO1-mediated canonical signaling pathway. *FOXL2,* which encodes a forkhead transcription factor, was implicated in the repression of mammalian testis determination after a mutation at the goat locus was implicated in the etiology of XX maleness in the polled-intersex syndrome.^84^ *Foxl2* is expressed in a female-specific fashion in the mouse gonadal soma from around E11.5,^85^ but its expression is maintained in XX gonads lacking *Wnt4* and *Rspo1*,^79^,^86^ suggesting that it does not act downstream of the canonical WNT signaling pathway and may act autonomously in ovary determination. Initial studies of mice lacking *Foxl2* revealed a requirement for ovarian follicle formation: granulosa cell differentiation was abnormal, oocyte growth was impaired, and XX females were sterile due to oocyte atresia and progressive follicular depletion.^87^,^88^ Further analysis revealed that *Sox9* was highly upregulated in *Foxl2* −/− ovaries between birth and 1 week postpartum.^89^ Immunohistochemical studies indicated nuclear SOX9 expression in epithelial cells of *Foxl2*-deficient ovaries, indicating a switch from FOXL2 to SOX9 expression. Upregulation of additional testis-determining genes in supporting cells suggested derepression of the male pathway. Moreover, mutant ovaries contained cords with male-specific features; mutant supporting cells ranged in morphology from typical granulosa to Sertoli-like cells. These studies suggest an ongoing requirement for FOXL2 in the ovarian soma throughout development and maturation. Indeed, inducible deletion of *Foxl2* in adult ovarian follicles results in upregulation of testis-specific genes such as *Sox9* and reprogramming of granulosa and theca cells into Sertoli-like and Leydig-like cells.^90^ Thus, FOXL2 actively maintains an ovarian phenotype throughout life by mechanisms that appear to include the direct repression of *Sox9* transcription through the TESCO enhancer, in cooperation with estrogen receptors.^90^ Such antagonism, if mutual, would explain the exclusive expression of either FOXL2 or SOX9 at the cellular level observed in different contexts,^24^,^90^ though not all.^53^ However, as with *Wnt4* and *Rspo1*, absence of *Foxl2* does not lead to an early, embryonic form of sex reversal affecting the fate of germ cells.

Loss of both *Foxl2* and *Wnt4* resulted in newborn ovaries filled with testis-like tubules strongly expressing SOX9 and AMH.^86^ In the ovarian medulla, these tubules contained differentiated spermatogonia, indicating that, unlike single *Wnt4*- and *Foxl2*-deficient gonads, sex reversal in double knockout gonads extended to germ cells. Expression profiling revealed the upregulation of other testis-specific genes, including *Cyp26b1*, which antagonizes germ cell meiosis by degradation of retinoic acid (RA).^91^,^92^ Germ cell sex reversal indicates that the onset of sex reversal in double knockout XX gonads is much earlier than in the single gene knockout described earlier, although it falls short of the complete and early embryonic gonadal sex reversal observed in cases of transgenic XX gonads expressing *Sry* or ectopic *Sox9*.^47^,^48^,^93^ The picture emerging of ovary determination in mice is of quasi-autonomous, partially redundant modules that act to inhibit testis determination and activate ovarian genes: Z turns out to be genetically complex. Indeed, novel ovary-determining genes/modules may yet remain to be identified, but we await the phenotype of XX mouse gonads lacking all of *Rspo1*, *Foxl2*, and *Wnt4* before concluding that other compensating pathways exist. In higher mammals, such as sheep and humans, these modules may be more closely integrated and interdependent, resulting in female-to-male gonadal sex reversal when only individual genes are disrupted.^78^,^84^,^94^ The fact that estrogens do not appear to function in female embryonic development in the mouse may also account for such species differences in the response of the gonad to FOXL2 deficiency, given the role of estrogen receptors in antagonizing TESCO in the adult ovary.

In *Foxl2*-deficient ovaries, testis-like somatic differentiation occurred in the presence of oocytes that progressed through meiotic prophase with no morphological abnormalities.^89^ Several studies have suggested that oocytes are required to maintain female somatic differentiation,^95^,^96^ but oocyte degeneration would not appear to be an explanation of somatic cell sex reversal in *Foxl2*-deficient XX gonads. Direct ablation of oocytes in postnatal and 8-week-old ovaries did not result in transdifferentiation of granulosa cells into Sertoli-like cells, and thus oocyte loss does not account for the somatic transdifferentiation observed after deletion of *Foxl2* in adult ovaries. However, in other experimental contexts, such as when fetal ovaries are grafted to the kidney capsule, freemartin cattle, irradiated neonatal rats, and transgenic mice overexpressing AMH, loss of oocytes has been thought to underlie the appearance of Sertoli-like and Leydig-like cells.^96^–^98^ It is unclear how these disparate observations can best be reconciled. The timing of germ cell loss in these models appears to be critical; XX gonads that have never contained oocytes due to early loss or absence of germ cells are not disposed to develop Sertoli-like cells.^94^ It has been proposed that only loss of meiotic germ cells during a window of opportunity, when granulosa cells have the competence to acquire Sertoli-like characteristics, can result in transdifferentiation.^96^ In this context, it is interesting to note that genes associated with early testis differentiation are activated late in gestation and during the immediate postnatal period in the mouse ovary, possibly associated with ovigerous cords at this stage.^99^ The expression of protestis genes might confer additional plasticity on the granulosa cell lineage. Moreover, molecular alterations to somatic cells may even prefigure oocyte loss in some of these aforementioned models, in a similar fashion to loss of oocytes caused by deletion of ovarian somatic genes, such as *Wnt4* and *Fst*.^77^ Whatever the role for oocytes in maintenance of ovarian somatic cell identity, oocytes can antagonize testis cord formation in organ culture models.^100^ This effect of oocytes might account for the delayed sex reversal in *Foxl2*-deficient ovaries. However, the action of additional ovary-determining genes, such as GATA4 and ZFPM2,^101^ at earlier stages cannot be excluded. For a discussion of germ cell sex determination itself, see Box 2.

**BOX 2 GERM CELL SEX DETERMINATION**

In addition to being endocrine glands, the gonads function as factories for the production of haploid gametes that are required for sexual reproduction. There has been major progress recently in our understanding of the control of the fate of primordial germ cells, which enter the newly formed mouse gonad from around E10.5. It has been known for many years that germ cells are bipotential in the early stages of gonad development: XY germ cells can enter the pathway of oogenesis and XX germ cells can similarly initiate spermatogenesis. Examples of this are given in the main text and are usually associated with sex reversal of the gonadal soma, but analysis of XX-XY chimeras has also established this bipotentiality.^69^,^95^ Thus, it is not the chromosome constitution of primordial germ cells that determines their initial fate but the gonadal environment in which they develop.^102^ In the developing mouse ovary, germ cells cease mitosis at around E12.5–13.5 and enter meiosis. In contrast, germ cells in the developing testis arrest in G0/G1 phase of mitosis, and these gonocytes remain in this quiescent state until after birth. A number of experiments had led to the postulation of a meiosis-inducing substance: XY germ cells can be ‘coaxed’ into meiosis if male gonads are cultured with conditioned media from cultured ovaries.^103^,^104^ Indeed, a substance secreted from the rete ovarii was a candidate for such a meiosis inducer.^105^ The conclusion was that male germ cells are somehow protected from the influence of this inducer of meiosis.

It is now clear from a number of studies that RA, thought to be produced from the adjacent mesonephros, is responsible for inducing meiosis in the developing mouse ovary and that germ cells in the XY gonad are protected from effects of RA through the production of high levels of the P450 enzyme CYP26B1 by Sertoli cells.^91^,^92^ CYP26B1 acts to degrade RA and in XY gonads lacking the *Cyp26b1* gene XY germ cells enter and progress through meiosis in an otherwise normal testicular environment, before being lost by birth through apoptosis.^106^ Loss of *Cyp26b1,* as late as E15.5, can cause XY germs cells to enter meiotic prophase.^107^ Culturing the fetal testis in the presence of exogenous RA can induce XY germ cells to enter meiosis, indicating that RA acts directly on germ cells.^91^,^92^ Earlier observations that germ cells colonizing locations outside the developing gonads spontaneously enter meiosis are perhaps best explained, not by the existence of some default meiotic pathway but by the relative ubiquitousness of RA in the embryo (reviewed in Refs 108 and 109).

However, entry into the male pathway of germ cell development is not a mere default response to the absence of RA signaling. Recent studies show that the secreted growth factor FGF9 does not just act in concert with SOX9 to drive the differentiation of Sertoli cells; it also acts directly on germ cells in the XY gonad to inhibit meiosis, maintain expression of pluripotency markers, and upregulate markers of male germ cell fate.^66^ Thus, our current understanding of germ cell fate commitment mirrors that of the gonadal supporting cell lineage, involving the mutually antagonistic action of molecules (CYP26B1/FGF9 vs RA) that act to control commitment to germ cell mitotic arrest or meiosis. One other observation can be made that relates somatic cell fate to germ cell fate: it seems likely that one consequence of the envelopment of germ cells by Sertoli cells and peritubular myoid cells during testis cord formation is the protection of XY germ cells from the effects of mesonephric RA. The conclusion that this consequence is an adaptation leads to the idea that a defining event in testicular morphogenesis, cord formation initiated by SRY, occurs in order, at least in part, to ensure appropriate germ cell sex determination.

## DISORDERS OF SEX DEVELOPMENT (DSD) IN HUMANS

### 46,XY DSD

Abnormalities of human sexual development, gonadal or genital, are relatively common, occurring in approximately 1 in 250 newborns. DSD have been defined as congenital conditions in which development of chromosomal, gonadal, or anatomical sex is atypical.^110^ Individuals with 46,XY DSD may exhibit a range of pathologies, including ambiguous genitalia, penoscrotal hypospadias, cryptorchidism, dysgenetic testes, reduced sperm production, and persistent Müllerian duct derivatives.^111^ 46,XY complete (or pure) gonadal dysgenesis (46,XY CGD) is much rarer than 46,XY DSD and is characterized by a 46,XY karyotype with normal female genitalia and completely undeveloped, fibrous (‘streak’) gonads. This latter class is likely to represent instances of early gonadal sex reversal in which ovarian morphology is subsequently disrupted, purportedly due to loss of germ cells. 46,XY ovotesticular DSD, formerly 46,XY true hermaphroditism, is characterized by the presence of testicular and ovarian tissue in the same gonad.

Approximately one third of 46,XY CGD cases are caused by point mutations or deletions of *SRY*.^112^ Other genes associated with spontaneous or familial cases of 46,XY DSD and CGD include *NR5A1*,^113^ *DHH*,^114^ *NR0B1*,^115^ *WNT4*,^116^ *CBX2*,^117^ *DMRT1* and *DMRT2,*^118^,^119^ and *MAP3K1*.^120^ In the case of *NR0B1* and *WNT4*, rare gene duplications are thought to cause the XY gonadal abnormalities. Dosage-sensitive sex reversal (DSS), an XY sex reversal phenomenon in humans, is thought to be due to duplication of *NR0B1*, although *Nr0b1* is also required for testis determination in mice on specific genetic backgrounds and not ovary development^121^; this suggests a complex, dosage-sensitive role for this protein in mouse and human sex determination.^15^,^122^

Mutations causing both 46,XY GD and additional congenital abnormalities have been described in *WNT4*,^123^ *NR5A1* (adrenal failure and XY sex reversal),^124^,^125^ *WT1* (Frasier syndrome),^126^ *SOX9* (campomelic dysplasia),^44^,^45^ *ATRX* (*α*-thalassemia/ mental retardation/gonadal dysgenesis),^127^ and *GATA4* (heart defects and GD).^128^ However, around two thirds of instances of 46,XY CGD and 46,XY partial GD remain to be explained at the molecular level, suggesting that notwithstanding the possibility of undetected regulatory mutations in known sex-determining genes, novel human testis-determining loci are yet to be identified. Many of the genes listed above are sex determining in the mouse, but not all, raising the possibility of species-specific differences in eutherian mammals with respect to testis determination and differentiation. For example, *ATRX* mutations cause urogenital abnormalities including testicular dysgenesis and female or ambiguous external genitalia.^129^,^130^ In mice, when *Atrx* is deleted by E14.5 in the developing Sertoli cells, mutant testes were smaller by late gestation due to proliferative defects and apoptosis but external genitalia were normal.^131^ Even when pathways appear to be conserved, differences between species may arise. For example, *Map3k4* is known to be required for mouse testis determination, while mutations in the closely related gene, *MAP3K1,* are associated with 46,XY CGD in humans.^30^,^120^ *Map3k1* is not, however, required for mouse testis determination.^132^ Details of the mechanistic role of MAPK signaling in testis development should solve the problem of apparent discrepancies such as this and reveal whether the bigger picture is one of conserved or divergent mechanisms. It is also worth noting that loci implicated in the regulation of *Sry* expression in mouse are common in the list of human genes above, and these, plus unidentified regulators, may underlie a large proportion of cases of 46,XY DSD and CGD not caused by disruption to *SRY* itself. Certainly, factors required for specification of Sertoli cell fate, or implicated in antagonizing this when duplicated, appear to comprise the bulk of the associated loci.

### 46,XX Testicular DSD

46,XX testicular DSD is characterized by a 46,XX karyotype with male genitalia ranging from normal to ambiguous, the presence of testes, azoospermia, and absence of Müllerian structures.^133^ The presence of the *SRY* gene accounts for approximately 85% of cases of XX testicular DSD. *SRY*-negative XX testicular DSD is a very rare but fascinating genetic phenomenon that suggests (1) the existence of a genetic suppressor of male development acting during ovary development^74^ and (2) genes that can initiate testis development in the absence of *SRY* (see *Ovary-Determining and Antitestis Genes*). Mutations of *RSPO1* associated with XX testicular DSD, palmoplantar hyperkeratosis, and predisposition to squamous cell carcinoma were reported in a consanguineous family and a sporadic case.^78^ These mutations are thought to be loss-of-function alleles. In addition to male or ambiguous external genitalia and hypogenitalism, gonadal biopsies revealed the presence of testicular structures and ovotesticular morphology (strictly 46,XX ovotesticular DSD) in affected XX individuals.^134^,^135^ These data establish *RSPO1* as a human ovary-determining gene with antitestis functions, in line with the ‘Z’ hypothesis discussed above.

In addition to loss-of-function mutations, gain-of-function of testis-determining genes, or genes with the ability to act ectopically as such, including *SOX9* and *SOX3*, is also associated with XX testicular DSD.^10^,^49^,^50^

## CONCLUSIONS

We have seen that sex determination in mice and humans is characterized by the operation of mutually antagonistic gene regulatory networks. Testis determination is initiated by *SRY* expression, but this masculinizing signal must spread throughout a long, thin primordium. Failure to do so in a timely fashion can result in XY ovotestis development or complete sex reversal, depending on the degree of disruption. The ‘battleground’ for these antagonisms is the supporting cell precursor, and the upshot of the ‘battle’ is a canalized developmental pathway of organogenesis. Moreover, the molecular antagonisms crucial to fetal sex determination appear to operate continuously in maintaining the differentiated state of the adult gonad, based on gene deletion studies in mice. The role of a range of sex-determining genes in maintenance of the adult gonadal soma (and germ cell lineage) will no doubt be a continued research focus.

Many questions remain concerning the regulation of gonad formation and morphogenesis: these concern the control of gonad size and growth, of the precise origin of specific cell lineages such as the peritubular myoid and fetal Leydig cells, and the regulation of the number of testis cords in males. In females, the quasi-autonomous modules that currently dominate our thinking about ovary determination must be understood in more detail, such that the plasticity of the ovarian soma and any reliance on germ cells for its stability is clarified. Most importantly, we cannot profess to any real understanding of how the myriad cellular processes comprising gonad development, especially embryonic testis development, are integrated and coordinated. Moreover, molecular details concerning the regulation of key genes, such as *SRY*, *FGF9* and *FOXL2*, are scant, as are details of the targets of numerous transcription factors. The molecular bases of the genetic antagonisms described here also await detailed characterization.

Given the major role played by positional cloning of human DSD/GD loci in identifying novel mammalian testis-determining genes hitherto, the advent of new genomic and epigenomic technologies is likely to mean the identification of more sex-determining genes in humans.^136^ Studies in the mouse, meanwhile, may also contribute genetic and phenotypic novelty by the use of forward genetic screens^30^ and will continue to allow detailed investigation of molecular and cellular mechanisms in mammalian sex determination and differentiation. Advances in live imaging, microscopy, conditional gene ablation, and novel genetic approaches to overcoming functional redundancy are also likely to shed more light on gonadogenesis and the mechanistic bases of gonadal sex reversal.
